# Model-based experimental manipulation of probabilistic behavior in interpretable behavioral latent variable models

**DOI:** 10.3389/fnins.2022.1077735

**Published:** 2023-01-09

**Authors:** Janine Thome, Mathieu Pinger, Daniel Durstewitz, Wolfgang H. Sommer, Peter Kirsch, Georgia Koppe

**Affiliations:** ^1^Department of Theoretical Neuroscience, Central Institute of Mental Health, Medical Faculty Mannheim, Heidelberg University, Mannheim, Germany; ^2^Department of Psychiatry and Psychotherapy, Central Institute of Mental Health, Medical Faculty Mannheim, Heidelberg University, Mannheim, Germany; ^3^Department of Clinical Psychology, Central Institute of Mental Health, Medical Faculty Mannheim, Heidelberg University, Mannheim, Germany; ^4^Faculty of Physics and Astronomy, Heidelberg University, Heidelberg, Germany; ^5^Institute of Psychopharmacology, Central Institute of Mental Health, Medical Faculty Mannheim, Heidelberg University, Mannheim, Germany; ^6^Institute of Psychology, Heidelberg University, Heidelberg, Germany

**Keywords:** reward discounting, delay discounting, computational models, behavioral model, design optimization, adaptive design, homogenizing behavior, computational psychiatry

## Abstract

**Introduction:**

Interpretable latent variable models that probabilistically link behavioral observations to an underlying latent process have increasingly been used to draw inferences on cognition from observed behavior. The latent process usually connects experimental variables to cognitive computation. While such models provide important insights into the latent processes generating behavior, one important aspect has often been overlooked. They may also be used to generate precise and falsifiable behavioral predictions as a function of the modeled experimental variables. In doing so, they pinpoint how experimental conditions must be designed to elicit desired behavior and generate adaptive experiments.

**Methods:**

These ideas are exemplified on the process of delay discounting (DD). After inferring DD models from behavior on a typical DD task, the models are leveraged to generate a second adaptive DD task. Experimental trials in this task are designed to elicit 9 graded behavioral discounting probabilities across participants. Models are then validated and contrasted to competing models in the field by assessing the ouf-of-sample prediction error.

**Results:**

The proposed framework induces discounting probabilities on nine levels. In contrast to several alternative models, the applied model exhibits high validity as indicated by a comparably low prediction error. We also report evidence for inter-individual differences with respect to the most suitable models underlying behavior. Finally, we outline how to adapt the proposed method to the investigation of other cognitive processes including reinforcement learning.

**Discussion:**

Inducing graded behavioral frequencies with the proposed framework may help to highly resolve the underlying cognitive construct and associated neuronal substrates.

## Introduction

Behavioral latent variable models which describe an individual’s trial-by-trial behavior in terms of well interpretable generative equations have become increasingly popular in neuroscience and psychiatry to quantify the mechanisms underlying cognitive processes involved in decision-making (Durstewitz et al., 2016; Huys et al., 2016). By inferring such models from an individual’s choice sequence recorded during an experiment, the underlying cognitive processes which echo in these choices can be mapped onto an often low-dimensional set of interpretable model parameters, the involved sub-functions can be teased apart, and hypotheses directed at the algorithmic principles of the given process may be addressed (e.g., Huys et al., 2013; Collins et al., 2017; Koppe et al., 2017; Thome et al., 2022).

Besides these clear advantages, as of yet, one important aspect of such models has often been overlooked. Since they attempt to fully explain trial-by-trial behavior, these models typically incorporate all relevant factors necessary to describe variations in behavior. This also means that they (implicitly) predict how behavior would change if any of the relevant factors is varied. On the one hand, such model-based predictions can be leveraged to steer or *induce* behavior by manipulating the experiment (by varying one or more of the above-mentioned relevant factors), thus providing a formal recipe to generate adaptive model-based experiments (Thome et al., 2022). On the other hand, by comparing a broad range of these predictions to actual behavioral observations, we obtain a formal framework ideally suited to validate a given model. Here, we therefore build on a previously introduced generic model-based framework to improve the generation of adaptive experiments (Thome et al., 2022), and couple it to a formal behavioral model validation procedure.

We have illustrated the procedure in the context of delay discounting. Delay discounting refers to the tendency of an individual to favor immediate as compared to temporally distant outcomes due to future outcome devaluation. Since individuals differ strongly in their discounting behavior, adaptive tasks which aim at adjusting trials to the individual to induce and measure more homogeneous discounting behavior, have been the means of choice for quite some time (Monterosso et al., 2007; Ripke et al., 2012; Cavagnaro et al., 2016; Koffarnus et al., 2017; Pooseh et al., 2018; Ahn et al., 2020; Knorr et al., 2020). In a typical delay discounting task such as the intertemporal choice task (ICT), participants are faced with a series of choices between a delayed larger and immediate smaller reward (e.g., Mazur, 1987). A common model of behavior in the ICT assumes that choices are probabilistic draws based on internal choice values with a higher likelihood for choices of higher value (e.g., Pine et al., 2009; Prevost et al., 2010; Miedl, 2012; Peters et al., 2012; Ahn et al., 2020). These choice values are computed based on the presented rewards and delays in the experiment, the discounting function, and individual-specific discounting parameters which regulate its behavior. A probabilistic function maps these latent values to probabilities for immediate and delayed choices. By setting the conditional probability for an immediate choice to a given response probability for each unique participant, we can resolve the model equations for a condition that expresses how experimental stimuli need to be selected so that we can expect to observe this response probability. For example, when setting the discounting probability in an ICT to 0.5, this condition will express how to adjust rewards and delays in a given participant to obtain 50% discounted choices. We have recently successfully applied this framework to induce a 0.3, 0.5, and 0.7 discounting probability across individuals (Thome et al., 2022).

On the other hand, manipulating the experimental variables simultaneously renders *predictions* over behavioral response probabilities for a given model. The fields of statistical learning theory and machine learning (ML) instruct us on how to make use of such predictions to objectively assess model validity (Hastie et al., 2009; Koppe et al., 2021). Validity here refers to whether a function – for instance, a statistical model – generalizes well to the population and has a low expected prediction error (PE; Hastie et al., 2009; Koppe et al., 2021). In short, a method or function is valid if we can infer it on a sample and use it to predict new unseen measurements with low error (Hastie et al., 2009). This corresponds well to the psychological perspective on validity by which validity denotes the extent to which evidence and theory justify the interpretation of measurements (Schmidt-Atzert and Amelang, 2021). The appealing part about assessing a PE is that it provides an objective way to assess predictive validity, and may further yield quantitative information on how and where (i.e., in what domain) a method is valid. Here, we thus extend our model-based adaptive approach to (a) predict and induce a wider range of behavioral response probabilities, and (b) use these predictions to perform a formal assessment of the PE.

The advantages of such a procedure are manifold. For one, the approach provides a recipe of how to generate adaptive experiments that ensure similar behavioral probabilities between participants. Effectively, such a procedure reduces behavioral variance within experimental conditions and thereby increases statistical power (Winer, 1971; Mumford, 2012). At the same time, the proposed procedure relocates between-subject variability into the adaptive experimental variables and model parameters, such that this information is preserved and can be systematically studied (Kanai and Rees, 2011; Hedge et al., 2018). Second, by generating experimental conditions which induce graded response probabilities, we may also induce graded intensities of the underlying process, resolving it at a finer level. This is beneficial when linking behavior to, for instance, neuro(physio)logical mechanisms (Dagher et al., 1999; Grinband et al., 2006; Wood et al., 2008; Hare et al., 2009; Ripke et al., 2014; Grosskopf et al., 2021). Finally, the formal assessment of model validity in terms of (out-of-sample) estimates of the PE allows us to compare and select between a class of available or novel models, and evaluate the models on a wide range of the model domain.

The present work illustrates this procedure in the context of monetary reward delay discounting, and expands it to a broader class of cognitive domains. We address the hypothesis whether by applying the proposed approach we can successfully induce (relative) discounting frequencies on a 9-level graded scale (ranging from 0.1 to 0.9) which, to the best of our knowledge, has never been attempted before. We then illustrate how to formally assess (predictive) model validity within this framework by comparing predicted to induced response frequencies, and evaluate several models on a group and single-subject level. Finally, we outline how to adapt the approach to latent variable models which are history dependent as well as response models which are multi-categorical.

## Materials and methods

### Experimental design

#### General framework

The key aspect of the proposed framework is to experimentally manipulate the latent process of a latent variable model, and thereby generate precise and falsifiable hypotheses about the data generating process (and associated cognitive functioning) in a systematic manner. These hypotheses (i.e., predictions) are statements about the frequency of observed responses in consequence of the experimental manipulation. Latent variable models which formalize the latent process and its dependencies on experimental variables, and probabilistically link this process to behavior, provide the means for such a manipulation. This is because these models let us track how changes in the experimental variables will affect behavioral probabilities. By making use of this property, we can systematically tune the experiment to generate a given behavioral probability.

**FIGURE 1:**
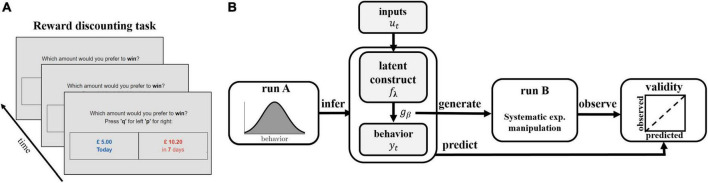
Schematic illustration of task and experimental framework. **(A)** Illustration of the reward discounting task. Participants are faced with a series of binary choice trials, in which they are asked to choose between an immediate smaller reward and a delayed larger reward. **(B)** Illustration of experimental protocol. Participants perform run A of the reward discounting task. Latent discounting models *f*_*λ*_ are inferred on each participant’s sequence of observed behavioral choices *Y* = {*y*_1_,…,*y*_*T*_} in run A and used to generate trials of run B. Trials are systematically manipulated by varying experimental inputs *u_t_* to induce discounting frequencies ranging from 0.1 to 0.9, based on the expectation of the probability distribution *g*_*β*_. Validity is assessed by comparing predicted and observed rel. choice frequencies.

The framework proceeds in two experimental runs (see Figure 1). The first run (termed ‘run A’) serves to generate data to infer the models and thus the latent process of interest. The models are then leveraged to generate predictions and associated experimental manipulations which are then assessed in a second run (termed ‘run B’). By separating run A and B in this way, we ensure that the trial-generation procedure is not biased by type of model applied, that is, the model is not inferred on trials it has itself selected. Validity of the instrument is measured by comparing these predictions to observations made in run B (Figure 1B). We illustrate and evaluate the framework here based on the case where we have a latent variable model with no history dependence combined with a binary response model. A transfer of the proposed approach to latent variable models with history dependence and response models which are multi-categorical is found in the Results section.

#### Binary response models with no history-dependence

Delay discounting provides a prominent example of a binary choice process in which the latent process does not depend on history (i.e., each choice depends only on current and not previous choice values). In the delay discounting example, run A and run B consist of an ICT. In this task, participants are faced with a series of binary choice trials in which they are asked to choose between an immediate smaller reward and a delayed larger reward (see Figure 1A). The collected data set *d* thus consists of *T* pairs of *observed* variables*d* = {(*x*_*i*_,*y*_*i*_),*i* = 1,2,…,*T*}, where *x_i_* are predictor vectors of immediate and delayed reward and delay pairs, and *y_i_* are one-dimensional (dichotomous) observed responses of immediate (*y*_*i*_ = 1) and delayed (*y*_*i*_ = 0) choices. The sequence of observed choices *Y* = {*y*_1_,…,*y*_*T*_} is modeled as i.i.d. Bernoulli random variables *y*_*i*_∼*Bi*(1,μ(*x*_*i*_)), for *i* = 1,2,…,*T*, where μ(*x*_*i*_) is the probability of an immediate choice given the predictor*x_i_*.

The probabilistic latent variable discounting model estimates μ(*x*_*i*_) by mapping the observed predictor vectors *x_i_* onto the conditional mean of the distribution of *y_i_* via a latent process *f*_*λ*_, that is, $\hat{\mu }\left(x_{i}\right):=E\left[y_{i}|f_{\lambda }\left(x_{i}\right)\right]$. *f*_*λ*_ is a discounting function mapping observable predictors *x_i_* onto internally represented *latent* values *v_i_* of these predictors, and $\hat{\mu }\,\left(x_{i}\right)$ maps these values onto the conditional mean of the Bernoulli distribution.

As discounting model *f*_*λ*_, we chose a hyperbolic function with exponential delay termed ‘modified hyperboloid model’ in the following (after Mazur, 1987; Rachlin, 2006). The discounting model is a vector valued function mapping two rewards *r* presented at two delays *D* (displayed in each trial of the ICT and collected in predictors *x_i_* above) onto internally represented values *v* for the two associated choices.


(1) $$f_{\lambda }\left(r,D\right):=\left(\frac{1}{1+\kappa \cdot D^{s}}\right)r$$

where κ is a discount parameter capturing the individual tendency to discount, and *s* is a scaling parameter, both ⊂λ. In each trial of the ICT, the discounting model thus maps an immediate reward *r*_imm_ (presented at 0 delay) and a delayed reward *r*_*del*_ presented at delay *D_i_* onto immediate and delayed values *v*_imm_ and *v*_del_ for the respective choice. Since the immediate reward has 0 delay, it is equal to its latent value, i.e., *r*_*imm*_ = *v*_imm_. We will refer to the factor in front of *r* in the following as the ‘discount factor.’ We have previously shown that this model performs consistently better at predicting unseen behavior than a number of other models (Thome et al., 2022; see also Estle et al., 2006; Odum et al., 2006; Rachlin, 2006; Rodzon et al., 2011; McKerchar et al., 2013; Cox and Dallery, 2016; Białaszek et al., 2020).

**FIGURE 2:**
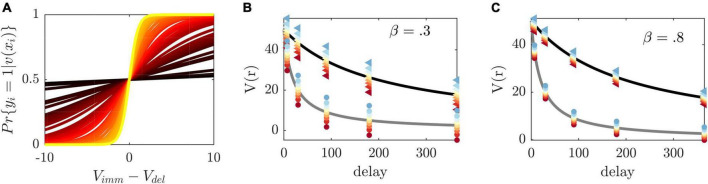
Illustration of method principles. **(A)** Immediate choice probability [cf. Eqn. (2)] as a function of the difference between immediate and delayed value for β estimates in our sample (color-coded from largest β=2 in yellow to smallest β=0.01 in dark red). The indifference point, i.e., the point at which immediate and delayed choice probability (and immediate and delayed choice values) are equal, is at 0. If *v*_*imm*_/*v*_*del*_ immediate choice probability is below/above 0.5. β regulates the steepness of the curve and thus the sensitivity toward differences in values. **(B)** Discounted value of a reward of size 50 (*y*-axis) delayed at different time points (*x*-axis) and two exemplary κ′s (κ=0.005 in black and κ=0.05 gray). The method’s selected immediate rewards at a given delay are displayed as colored dots from blue to red with respect to the induced choice probability from 0.1 to 0.9 (triangles/circles are associated with κ=0.005/0.05, respectively). To induce the same probabilities at different delays, the difference between the presented immediate choice values (depicted as colored dots) and delayed values (depicted on the discounting curve) is constant [see Eqn. (2), *v*_*del*_-*v*_*imm*_]. For participants with different κ, the reward and value ratios will therefore vary. The left graph depicts selected rewards for a hypothetical β=0.3 and **(C)** the right graph for β=0.8. β thus regulates the precise difference between immediate and delayed values, with higher β resulting in smaller differences, making the differentiation between the two more difficult.

We choose a sigmoid function to map these two latent values onto the conditional mean, that is, onto the probability for selecting the immediate choice option.


(2) $$\hat{\mu }\,\left(x_{i}\right)=\frac{1}{1+e^{\beta \,\left(v_{d\,e\,l}-v_{i\,m\,m}\right)}}$$

where β is an individual-specific parameter which captures the sensitivity to differences in choice values (see also Figure 2A). Eqn. (2) maps differences in values to immediate choice probabilities *p*_imm_ (see Figure 2A), in close analogy to a psychometric function (e.g., Wichmann and Hill, 2001). It provides a condition which permits the systematic manipulation of experimental conditions. By setting *p*_imm_ to a given probability, we obtain an equation which may be resolved for an observable and tunable experimental variable. For instance, solving Eqn. (2) for the immediate reward *r*_*imm*_ by plugging in the model assumptions, we obtain the following condition.


(3) $$r_{i\,m\,m}=\left(\frac{1}{1+\kappa \,D^{s}}\right)\,r_{d\,e\,l}+\frac{l\,o\,g\,\left(\frac{p_{i\,m\,m}}{1-p_{i\,m\,m}}\right)}{\beta }$$

defined for 0< *p*_*imm*_ < l. By inserting a set of fixed delays and delayed rewards, as well as inferred subjective parameters κ and β, Eqn. (3) expresses how to experimentally manipulate the presented immediate reward to obtain a desired immediate choice probability *p*_*imm*_ in a given individual (please see Figures 2A–C on operating principles of the method). By aiming to construct experimental conditions with similar immediate choice probabilities across participants, we are effectively homogenizing behavior across participants. We make the implicit assumption here that behavior is homogeneous if, within an experimental condition, different participants display similar frequencies, that is, they show similar probabilities, for the available behavioral options.

For the conducted experiment, models inferred on run A were applied according to this framework to generate an ICT with nine experimental conditions inducing graded immediate choice probabilities of *p*_*imm*_ = {0.1,0.2,0.3,0.4,0.5,0.6,0.7,0.8,0.9} presented in run B (see Figure 1), for simplicity referred to as ‘induced frequencies’ in the following.

#### Experimental settings

Trials in run A followed a previously developed protocol optimized to elicit discounting behavior across participants (Thome et al., 2022) and optimized in line with results obtained from a preliminary experiment (see Supplementary Text 2). Rewards and delays varied across trials. Delays were set to *D* = {7, 30, 90, 180, 365} days and delayed rewards to *r*_*del*_ = {5, 10, 20, 50, 100}£ (UK). Immediate rewards were selected based on the described model guided procedure [Eqn. (3)], chosen to elicit an equal probability for immediate and delayed choices at 4 different population representative discounting parameters. Run A thus consisted of 100 trials (5 delays × 5 delayed rewards × 4 discounting parameters). Trials of run B were generated via Eqn. (3), and the parameters inferred on run A, to induce 9 probability gratings ranging from 0.1 to 0.9. With nine gradings and using the same delays and delayed rewards as in run A, run B consisted of 225 trials.

**FIGURE 3:**
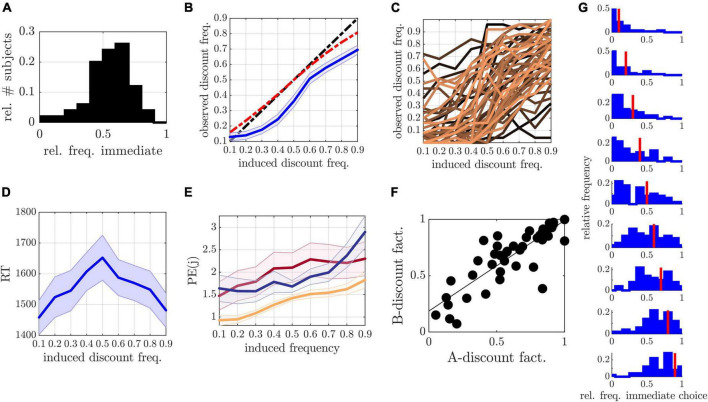
**(A)** Relative frequency of immediate choices in run A. **(B)** Relative frequency of discounted choices (*y*-axis) as a function of model induced frequencies (*x*-axis) averaged over all participants (mean and SEM are displayed in blue). The black dashed line marks the identity, while the red dashed line shows the actual predicted frequencies according to the models. **(C)** Single participant curves. **(D)** Mean and SEM of reaction time (RT) as a function of experimental conditions. **(E)** Mean and SEM of prediction error (PE) as a function of experimental conditions for modified hyperboloid (yellow), hyperbolic (red), and hyperboloid control (blue) models. **(F)** Discount factor in run A (*x*-axis) and run B (*y*-axis) illustrated for delay = 90, indicating a reliable estimate of discounting across runs. **(G)** Histograms of immediate choice behavior for 0.1 (top) to 0.9 (bottom) frequency conditions (conditions indicated by the red line).

A few parameter and stimulus constellations could result in immediate rewards smaller than 0, or equal immediate and delayed rewards. To avoid such trials, the delays (and corresponding immediate rewards) were iteratively adjusted in the trial-generating procedure until reaching a minimum of 1 or a maximum of 365 days. If still not resolved, negative immediate rewards were set to 1 penny, while immediate rewards equaling delayed rewards were reduced by 1 penny, respectively. This adaptation could result in a slight deviation of the induced frequencies (see Figure 3B, red line). Trials were self-paced, allowing for a maximum decision phase of 10 s, with a 1 s inter-stimulus-interval.

#### Model inference

Discounting models were inferred on run A and run B separately via maximum likelihood estimation (MLE). Given Bernoulli i.i.d. assumptions, the models’ likelihood is given by $p\,\left(Y|X,\theta \right)=\prod_{i=1}^{T}p_{\theta }\,\left(y_{i}|v\,\left(x_{i}\right)\right)$, where *p*_θ_(*y*_*i*_|*v*(*x*_*i*_)) is given by Eqn. (2) in case that *y_i_* refers to the immediate choice, and by 1 minus this probability for the delayed choice, respectively. Under inspection of the preliminary experiment (see Supplementary Text 2), parameters were constrained to βϵ[0.001, 2],*s*ϵ[0, 1], and κϵ[0, 1000], and optimization was performed using a Quasi-Newton algorithm (the limited-memory BFGS algorithm) implemented via the optimize.minimize() function from the SciPy library^1^, starting from multiple initial conditions.

### Sample and data assessment

Fifty healthy participants (24 males, 25 females, and 1 undefined) participated in the study, recruited via the following website: https://www.prolific.co. Participants were eligible if they were between the age of 18 and 65 with current residency in the United Kingdom (UK) and were reimbursed £7.50 per hour to participate in the study. Please see Supplementary Tables 1, 3 for more information on the sample.

All participants accessed the study through a link on the Prolific website. They completed a consent form, filled out sociodemographic information, and took part in run A of the experiment. After completing run A, the alcohol use disorder identification questionnaire (AUDIT; Babor et al., 1992) and the short version of the Barratt Impulsiveness Scale (BIS-15; Spinella, 2007) were filled out, immediately followed by run B of the experiment. The whole procedure took 28.4 (±8.39) minutes on average. The study was approved by the local ethics committee of the Medical Faculty Mannheim, University of Heidelberg (2019-633N).

### Data analysis

#### Measured variables

We assessed the frequency of discounted choices (that is, choices in favor of the objectively smaller outcome) and median reaction time (RT) across run A, and for all 9 probability gradings (i.e., experimental conditions) in run B, model parameters (i.e., β,κ,*s*) the discount factor(s), as well as total scores of AUDIT and BIS/BAS questionnaires.

#### Inferential statistics

The agreement between experimentally induced frequencies and observed behavioral frequencies was assessed via a general linear model (GLM) with induced frequencies as linear predictor variables. The hypothesized inverted U-functional relationship between induced frequencies and RT was assessed via a GLM with quadratic induced frequencies as curvilinear predictor variables (hypothesizing higher/lower RT toward more difficult/easy choices) in run B. We report t-statistics on the regression coefficients of these models.

#### Prediction error assessment

Predictive validity was assessed by approximating the PE using cross-validation (CV). Rooted in statistical learning theory (Hastie et al., 2009; Efron, 2021), the PE quantifies the error made when applying a prediction rule, here the statistical model, to unseen (out-of-sample) data. Assuming that the (*x*,*y*) data pairs in the ICT follow an (unknown) joint distribution *F*, the PE quantifies the error made when drawing a new pair with only the predictor variable *x* observed and predicting *y* with $\hat{\mu }\,\left(x\right)$ (cf. section “General framework”) based on the model. Given some loss function $L\,\left(y,\hat{\mu }\,\left(x\right)\right)$ which assesses the deviation between observation and prediction, the PE is assessed as the expected loss under *F*, i.e., $E\,r\,r=E_{F}\,\left\{L\,\left(y,\hat{\mu }\,\left(x\right)\right)\right\}$ (Hastie et al., 2009; Efron and Hastie, 2021). Since this expectation goes over all (*x*,*y*) pairs, this integral may not be computed directly, but is in practice often approximated by resampling methods such as CV. Using CV, here we approximate the PE by ${\hat{E\,r\,r}}_{C\,V}=\frac{1}{T}\,\sum_{i=1}^{T}L\,\left(y_{i},\hat{\mu }\,\left(x_{i}\right)\right)$, where the summation runs exclusively over data pairs observed in run B and the prediction is based on models inferred on run A [denoted ‘PE (run B)’], and vice versa [denoted ‘PE (run A)’]. As most appropriate for dichotomous data, we employ as error function the binomial deviance (Efron, 2021), given by $L\,\left(\mu ,\hat{\mu }\right):=$2{$\mu log\frac{\mu }{\hat{\mu }}+\left(1-\mu \right)log\left(\frac{1-\mu }{1-\hat{\mu }}\right)\}$, such that the PE was assessed as


(4) $${\hat{E\,r\,r}}_{C\,V}=\frac{2}{T}\,\sum_{i=1}^{T}\left\{y_{i}\,l\,o\,g\,\frac{y_{i}}{\hat{\mu }\,\left(x_{i}\right)}+\left(1-y_{i}\right)\,l\,o\,g\,\left(\frac{1-y_{i}}{1-\hat{\mu }\,\left(x_{i}\right)}\right)\right\},$$

and $\hat{\mu }\,\left(x_{i}\right)\in \left[0,1\right]$ was truncated to [1e-10, 1 – 1e-10] to avoid infinities.

Since the integral in the PE runs across all possible (*x*,*y*) pairs, sampling a broad range of data pairs in run B – as achieved here by including nine experimental levels – should improve the estimation of *Err* by ${\hat{E\,r\,r}}_{C\,V}$. It furthermore allows to dissect and examine the PE for different experimental conditions. PEs were computed for each participant (i.e., at fixed parameter values), and averaged over to obtain a population estimate.

#### Model comparisons

The two experimental runs and PE assessment allows the objective comparison of different models in their prediction ability. Several models which varied in the assumption about the computation of the delayed values (and therefore the latent variable model) were evaluated in terms of PE (see Supplementary Table 2). These included the common hyperbolic model (Mazur, 1987; Davison and McCarthy, 1988), the exponential model (Samuelson, 1937), the constant sensitivity (CS) model (Ebert and Prelec, 2007), the modified hyperboloid model used for trial-generation (Mazur, 1987; Rachlin, 2006), the quasi-hyperbolic model (Phelps and Pollak, 1968; Laibson, 1997), the (conventional) hyperboloid model (Loewenstein and Prelec, 1992; Green et al., 1994), the double exponential model (van den Bos and McClure, 2013), and a control model to the modified hyperboloid model with β in Eqn. (2) fixed to 1. Details on these models can be found in the Supplementary Table 2. In addition, to investigate the model fit on a single participant level, we counted the number of individuals best described by each model.

#### Behavioral homogeneity

Reductions in behavioral variation within experimental conditions (i.e., increase in behavioral homogeneity) was tested by comparing variances of immediate choice frequencies via *F*-Tests across conditions of run B between the experiment and the preliminary data reported in the Supplementary Text 2.

#### Test–retest reliability

Finally, test–retest reliability was assessed by correlating the inferred parameters β,κ,*s*, as well as the discount factor(s) across runs A and B via Pearson correlation coefficients. Elements greater than 3 scaled median absolute deviation away from the mean were removed for these analyses to avoid spurious correlations.

## Results

The experiment is divided into two runs, run A and run B, where trials of run B were generated based on models inferred on single participant behavior in run A. Run B trials were generated such as to induce nine levels of discounting probability, ranging from 0.1 to 0.9. Most of the following results therefore concentrate on analyzing the success of inducing these probabilities in run B.

In run A, we observed an average frequency of discounted choices of 54% (±16%; see Figure 3A). Only 4% of our sample showed less than 20% discounted choices, rendering good conditions for model parameters to converge (see also Supplementary Tables 1, 3 and Supplementary Text 1 for further information on effects of gender, or associations to subjective measurements and sociodemographic information).

### Inferential statistics

In run B, observed discounting frequencies increased with induced frequencies on a group and individual level [group level slope: *T*(7) = 13.91, *p* < 0.001; Figure 3B; individual slopes: *T*(49) = 16.51, *p* < 0.001; Figure 3C]. On average, the offset and slope parameters obtained from the GLM came close to what was theoretically expected by the models, with an observed average offset of −0.017 [±0.23; *T*(7) = −0.51, *p* = 0.63] and a slope of 0.80 (±0.34) (where the expected offset and slope lay at 0.07 and 0.84, see Figure 3B red line). Median RTs moreover followed an inverse quadratic curve [significance of inverse quadratic predictor within GLM: *T*(6) = 7.41, *p* < 0.001; Figure 3D] as hypothesized.

Examining test–retest-reliability, the parameters β,*s*, and the discount factor (evaluated at *D* = 90) were significantly correlated between runs (β: *r* = 0.60, *p* < 0.001; *s* 0.38, *p* = 0.006; discount factor: *r* = 0.85, *p* < 0.001; see also Figure 3F), but not κ (*r* = 0.23, *p* = 0.21). The lack in reliability of κ was likely due to intercorrelations between κ and *s* known for this model (run A: *r* = −0.47, *p* < 0.001; run B: *r* = −0.4, *p* = 0.005; see also Thome et al., 2022), which, however, do not affect reliability of the discount factors.

### Behavioral homogeneity

To investigate whether the experimental framework was able to reduce variance within the induced experimental conditions, we compared variances within conditions of run B to the preliminary experiment (see Supplementary Text 2). All variances were either lower than or similar to those in the preliminary experiment (please also see Figure 3G for choice frequency distributions). Significantly lower variances were observed at frequencies 0.3 and 0.7 [0.3: *F*(48,49) = 2.03, *p* = 0.015, 0.7: *F*(48,49) = 1.8, *p* = 0.044], as well as marginally lower at 0.1, 0.2, 0.6, and 0.9 [0.1: *F*(48,49) = 1.63, *p* = 0.091, 0.2: *F*(48,49) = 1.7, *p* = 0.067; 0.6: *F*(48,49) = 1.72, *p* = 0.062; 0.9: *F*(48,49) = 1.76, *p* = 0.051]. Collectively, these results suggest that the induction protocol generated graded behavior which centered (comparatively) narrowly around model predictions.

### Prediction error assessment

**FIGURE 4:**
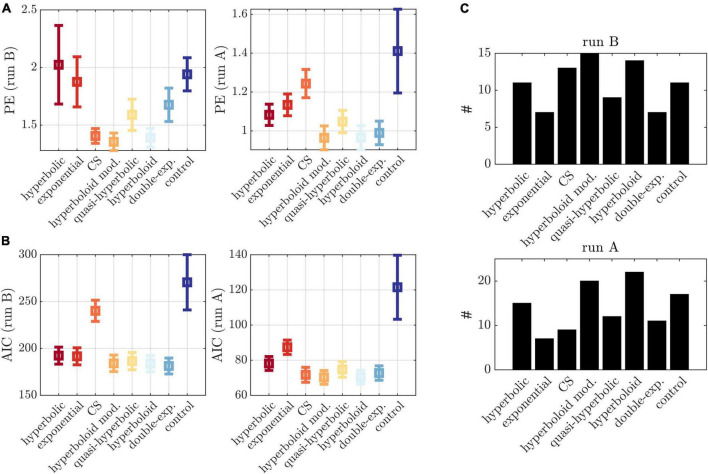
Model comparisons. **(A)** Left: PE in run B based on models inferred on run A for different models (*x*-axis). Right: PE in run A based on models inferred on run B for different models (*x*-axis). **(B)** AIC evaluated on run B (left) and run A (right). **(C)** Number of individuals (*y*-axis) with lowest PE for each model (*x*-axis) for run B (top) and run A (bottom), applying a tolerance threshold of 0.01. Cases with multiple minima were counted multiple times.

Corroborating these findings, we observed a comparatively low PE in run B for the applied modified hyperboloid models, that is, a low deviation between observed responses and responses predicted by the models inferred on run A (Figure 4A left). Statistically, the PE was lower than for the hyperbolic model (*p* = 0.046), the exponential model (*p* = 0.021), the double exponential model (*p* = 0.012), and the control model (*p* < 0.001) (and marginally lower than for the quasi-hyperbolic model; *p* = 0.084). This was largely consistent with the PE assessed on run A based on models inferred on run B (Figure 4A right), although here, the CS model performed significantly worse (*p* < 0.001), and the double-exponential model comparably (*p* = 0.173). These differences in the prediction ability of the evaluated models were not observed when applying the Akaike information criterion (AIC) as an in-sample error estimate of the PE (see Figure 4B), suggesting the AIC was less adequate to distinguish between models.

Interestingly, when examining the PE in the different experimental conditions of run B, we observed an increase in PE for higher induction frequencies (Figure 3E). Also, on an individual level, not all participants were best described by the hyperboloid models. In fact, we observed a wide spread over all models when counting the number of participants with lowest PE in each model (Figure 4C).

### Application to other latent variable models

Our framework to generate an adaptive experimental design was described and evaluated here based on the special case where the behavior generating model is characterized by a time-independent latent variable model and a simple binary response variable model. We therefore briefly outline here how to proceed when transferring the proposed framework to other cognitive functions and applications in which (a) the latent variable model is history-dependent (as for instance during learning), or (b) the response variable model is multi-categorical (as in tasks with more than two response options).

#### History-dependent latent variable model

We will first consider the case in which the computation of values within the latent variable model depends on previous values and is thus history dependent. As a simple example, we assume to be learning values toward two stimuli *u_1_* and *u_2_* via a Rescorla–Wagner type model in which our latent variable model *f*_*λ*_ now describes the formation of associative memory traces (=values) in time as a function of the reward prediction error,


(5) $$f_{\lambda }\,\left(u_{i}\right):=v_{t}\,\left(u_{i}\right)=v_{t-1}\,\left(u_{i}\right)+\lambda \,\left(r_{t}\,\left(u_{i}\right)-v_{t-1}\,\left(u_{i}\right)\right)$$

where λ is a learning rate parameter, *r*_*t*_(*u*_*i*_) is a reward or outcome associated with choosing the respective stimulus *u*_*i*_,*i* = {1,2},at time *t*, and *v*_*t*−1_(*u*_*i*_) is its prediction (Rescorla and Wagner, 1972). Eqn. (5) comes down to a recursive relationship in time which we can expand to its initial value:


(6), $$v_{t}\left(u_{i}\right)=\left(\lambda \sum_{n=0}^{t-2}\gamma^{n}r_{t-n}\left(u_{i}\right)\right)+\gamma^{t-1}v_{1}\left(u_{i}\right)$$

whereγ:=1-λ

For the response model, we select between these two stimuli such that we again end up with a Bernoulli process. To obtain a condition for generating adaptive trials which will induce a desired probability for selecting *u_1_* at time *t*, denoted here by *p_1_* (in analogy to selecting the immediate choice with probability *p*_*imm*_), we need to insert Eqn. (6) into a sigmoid such as in Eqn. (2), and then solve for *p_1_*. If, for simplicity, we further define $c_{i}:=\lambda \,\sum_{n=1}^{t-2}\gamma^{n}\,r_{t-n}\,\left(u_{i}\right)$ (which collects the history of rewards obtained for selecting stimulus *i*), we obtain the following trial-generating condition for this history dependent model:


(7) $$r_{t}\,\left(u_{1}\right)=\frac{l\,o\,g\,\left(\frac{p_{1}}{1-p_{1}}\right)}{\lambda \,\beta }+r_{t}\,\left(u_{2}\right)+\frac{c_{2}-c_{1}+\gamma^{t-1}\,v_{1}\,\left(u_{2}\right)-\gamma^{t-1}\,v_{1}\,\left(u_{1}\right)}{\lambda }$$

Eqn. (7) also makes sense intuitively. If we consider no prior knowledge [i.e., *v*_1_(*u*_*i*_) = 0] and no reward history (i.e., *c*_*i*_ = 0) and aim at generating trials which induce equal response probabilities for both options, we need to equalize the two rewards. If, in contrast, we have a higher initial value for selecting stimulus 2, we will need to add reward to stimulus 1. Finally, if we have already observed multiple rewards, the initial values will lose and reward history (reflected in *c_i_*) will gain importance. Such adaptive approaches may prove particularly suitable to address and control inter-individual variability in memory formation (e.g., Lonsdorf and Merz, 2017), and serve as an effective alternative (or addition) to threshold-based adaptation procedures.

#### Multi-categorical response model

In the second case, we consider a history-independent latent variable model coupled to a multiple choice response model. In such a case, the response probability *p_k_* of a response *y*_*k*_,*k* = 1,…,*K*, can be modeled in terms of a softmax function as $p_{k}=\frac{e^{\beta \,v_{k}}}{\sum_{i}e^{\beta \,v_{i}}}$, and the likelihood function will now follow a multinomial distribution. If we want to generate trials which will induce predetermined probabilities *p_k_* for response options *y_k_* with associated values *v_k_*, we therefore obtain the trial generating condition(s)


$$v_{k}=\frac{l\,o\,g\,\left(\sum_{j\neq k}^{K}e^{\beta \,v_{j}}\right)}{\beta }+\frac{l\,o\,g\,\left(\frac{p_{k}}{1-p_{k}}\right)}{\beta }.$$

Since in this multi-categorical case, we aim at controlling the probability of all *K* options simultaneously, we will also need to solve these *K* equations simultaneously, for instance, by some form of constrained optimization.

## Discussion

A general challenge in psychological and other sciences is that we want to uncover processes that are not directly observable, also termed latent processes. We draw inferences on these processes by observing behavior. In this context, experiments serve to generate conditions, that is, experimental manipulations, which differentially engage the latent process and are hypothesized to manifest in behavioral differences which allows us to study its nature in more detail. To draw an accurate inference on the underlying process based on these manipulations, we need to rely on our experiment and the latent process model being valid.

In the current work, we propose a framework which leverages interpretable probabilistic behavioral latent variable models to guide experimental manipulations and address the assessment of validity. By expressing the process in relation to the experiment and linking it probabilistically to behavior, these models allow us to generate precise and falsifiable hypotheses, i.e., predictions, and tune experimental variables to address these hypotheses (Thome et al., 2022). Predictions in this context are formulated as observable behavioral response probabilities. Assessing the deviation of predictions from out-of-sample observations facilitates the objective assessment of predictive validity. Here we apply the proposed approach to predict and induce graded choice frequencies on an individual participant level.

We illustrate the procedure in the context of delay discounting. Delay discounting is an influential psychological process, related to a variety of different traits such as impulsivity (Keidel et al., 2021), self-control (Levitt et al., 2020), intelligence (Shamosh and Gray, 2008), socio-economic status (Kohler et al., 2022), or personality (Keidel et al., 2021). It measures the tendency of an individual to devalue distant as compared to close future outcomes (Ainslie, 1975; Frederick et al., 2002; da Matta et al., 2012). Overly steep discounting has moreover been used to explain maladaptive behavior in addiction (Rabin and O’Donoghue, 1999; O’Donoghue and Rabin, 2000) and alcohol risk (Kohler et al., 2022), serving as a biomarker for the disease (Story et al., 2014; Bickel, 2015; Bailey et al., 2021; Cheng et al., 2021). Delay discounting is therefore of wide interest to both psychology and psychiatry. The general principle of the proposed framework in the context of delay discounting is that since we can infer probabilistic models which formalize how rewards are discounted as a function of delays and rewards (the cognitive process), then if we know the function (by model inference), we may determine how to vary experimental components so as to influence behavior.

We applied the proposed model-based approach to invoke discounting probabilities ranging from 0.1 to 0.9 in an (online) monetary reward discounting paradigm in a sample of healthy individuals. In line with model predictions, we observed a continuous (mostly linear) average increase in discounting frequencies coinciding with induced frequencies. Analyses of mean RT – as indirect measure of processing time – supported this notion as it followed an inverse U-function with higher RT toward trials with induced frequencies close to 0.5. This is to be expected since trials which induce equal or close to equal probabilities for immediate and delayed options are more difficult and may thus require more processing time (e.g., Ratcliff and Rouder, 1998). We also observed high test–retest reliability for the discount factor, replicating previous findings (Thome et al., 2022).

The model-based framework was successful at significantly reducing between-subject variance within several of the manipulated experimental conditions. This was observed in terms of lower behavioral variability in run B compared to a preliminary experiment with similar settings (see Supplementary Text 2). Low variance within experimental conditions is a prerequisite to obtain high power in associated statistical tests (e.g., Winer, 1971). In that sense, the proposed framework may also be seen as a tool which converts inter-subject variability into homogeneous ‘treatment conditions,’ increasing statistical power of an experimental design (Winer, 1971; Jackson, 2011; Boslaugh, 2012). At the same time, it does not eliminate important between-subject variability *per se* (Hedge et al., 2018; Goodhew and Edwards, 2019). Rather, between-subject variance is systematically relocated and captured in the (interpretable) model parameters and experimental variations. Relationships of this between-subject variance to other variables such as brain mechanisms or societal factors can therefore be explored.

The main strengths of the present framework though are the possibility to induce graded levels of behavior and to formally validate the trial-generating model and related models which reflect variations of a latent process. Assessing graded levels of behavioral probability benefits the resolution of the cognitive process at a fine-grained level. This is because behavioral probabilities reflect the intensity by which a cognitive process is engaged (in this example, the strength of temporal discounting). By studying fine gradings of behavioral probability, we may study the process on a dimensional level from low to high intensity. These intensities may be related, for instance, to neuro(physio)logical recordings to map the finely resolved latent process onto neural mechanisms (e.g., Ripke et al., 2014; Grosskopf et al., 2021, p. 20; Batsikadze et al., 2022). This may be of particular importance to psychiatry, where we aim at slowly moving away from studying psychiatric entities to stratifying patients in terms of dimensional alterations in different functional domains (RDOC; Insel et al., 2010).

The validation of the framework is realized by the implementation of two consecutive experimental runs which permit an estimation of the PE by cross-validation. We exploited this arrangement to validate the employed model by assessing its PE and comparing it to several discounting models proposed in the literature. The closely related hyperboloid models and the constant sensitivity model generated particularly low average PEs whereas the most commonly applied hyperbolic and exponential models performed comparatively poorly (in line with previous observations; Thome et al., 2022).

A modified hyperboloid (control) model with choice parameter β fixed to 1 performed particularly poorly (see Figure 4A). This emphasizes the importance of tuning β to the individual participant for a valid behavioral induction protocol. As outlined in the Supplementary Text 2, recovering β with high precision comes at the cost of increasing trial numbers. This is in line with a recent study by Pooseh et al. (2018) which performed simulation analyses to illustrate that at least 50–120 iterations are necessary for parameters to converge to their true values even when using an adaptive model-based Bayesian delay discounting framework. It challenges recent methods which infer discounting parameters in very few trials. For instance, Ahn et al. (2020) proposed a method to infer hyperbolic discounting models in less than 10 trials. While the authors demonstrate remarkably high reliability in measuring κ, they acknowledge poor reliability in β. It remains unclear how recovering models on the basis of few trials affects validity of other adaptive model-based designs. Unfortunately, most studies which have developed adaptive designs do not provide direct evidence for model validity, that is, they do not directly report predicted and actually induced response frequencies, (Monterosso et al., 2007; Cavagnaro et al., 2016; Koffarnus et al., 2017; Pooseh et al., 2018; Ahn et al., 2020), making it difficult to draw conclusions on validity of the available methods more generally.

An interesting insight of the present study is that model validity decreased particularly around hard trials, which are the main target of most other adaptive delay discounting methods (e.g., Ripke et al., 2012; Ahn et al., 2020; Knorr et al., 2020), and around larger immediate choice frequencies. One possible explanation for PE increases around hard trials is that the slope of the probability curve is steepest around hard choices (see Figure 2A where *v*_*imm*_ = *v*_*del*_). Small biases in the inference of discounted values (e.g., due to biases in parameter estimates) have the largest effect on changes in immediate choice probabilities, possibly resulting in higher behavioral variability in these conditions. This once more emphasizes the importance of an unbiased valid recovery of model parameters.

While it remains unclear why higher frequencies were associated with a higher PE, the example shows how dissecting the PE may uncover domains at which a method is less valid. In fact, a particular advantage of the proposed adaptive approach is that it allows to systematically perturb the different factors relevant to a choice process, obtain model-based predictions for these perturbations, and then validate the model thereon. For instance, in the present example, one could analogously vary the delay period (rather than immediate reward), and – using the CV approach – formally validate a broad range of the domain the discounting function is defined on. Effectively, we can thereby improve the criterion we aim at predicting to assess predictive validity.

In sum, the model evaluation results illustrate how the framework may be leveraged to select among a set of available models delineating variations of a given process model and identify domains at which a model may fail based on out-of-sample approximations of the PE (Hastie et al., 2009; Efron, 2021; Koppe et al., 2021). In the same way it could be used to identify and validate novel models or, differentiate a given model to alternative models (implicating discriminant validity). Out-of-sample predictions are crucial for validation since in-sample error estimates (as still commonly applied) have repeatedly been shown to be strongly biased (Hurvich and Tsai, 1989; Kuha, 2004; Hastie et al., 2009). In the present context, the AIC did not discriminate well between models whereas the out-of-sample PE did.

The present study separates model inference which is always based on the same constant set of trials in each participant (run A), from model-based prediction and manipulation (performed on run B). This differs from iterative approaches – most often employed in psychophysics to identify a psychometric function – which generate successive trials online based on an underlying (often simple sigmoid) model which is assumed to be true (Leek, 2001; Shen and Richards, 2012; see also Pooseh et al., 2018). Such approaches are ill-suited to compare an applied model to related models since the model-based procedure already biases trial selection and may moreover result in unequal trials and trial numbers per participant. Biased trial selections may likely favor some models over others (Owen et al., 2021; see also pitfalls of successive procedures Leek, 2001; Shen and Richards, 2012).

Historically, psychophysics has originated in aspirations to identify objective ‘laws of nature’ which map physical objects to sensation, i.e., rules which are thought to apply to everyone such as the Weber–Fechner law or Steven’s power law (Weber, 1835; Fechner, 1860; Stevens, 1957). Physical properties of experimental stimuli are therefore also typically directly mapped to detection or discrimination probabilities without an additional subjective transformation in between. Although latent variable models have more recently been applied to detect inter-individual differences (e.g., Taubert et al., 2012; Chakroborty et al., 2021; Owen et al., 2021), they are typically not used to generate adaptive trials (although see Thomas et al., 2021). Evaluating among a larger class of different subjective models has moreover not been of primary concern.

The latter may be specifically relevant to scientific disciplines which focus on uncovering inter-individual differences in (subjectively modulated) cognitive processes such as in the field of psychiatry for instance (e.g., Kanai and Rees, 2011). Here the focus often lies on how individuals (differentially) learn, interpret, or attribute information and how these processes may be subjectively modulated or biased (e.g., Koppe et al., 2017). In the present study, inter-individual differences are also supported by the observation of a high spread in the assignment of different discounting models to the individual participants, indicating different participants may best be described by slightly different ways of assigning subjective value to delayed outcomes (see also Cavagnaro et al., 2016).

While illustrated here on monetary delay discounting, the proposed framework may be adopted to many other contexts. Other popular and widely applied examples of interpretable latent variable models are for instance variants of reinforcement learning (RL) models which formalize the latent ‘learning’ process (e.g., Durstewitz et al., 2016; Sutton and Barto, 2018), and drift diffusion models which formalize latent evidence accumulation (Ratcliff, 1978). We outline here how to proceed in the case of reinforcement learning where latent variable models are history-dependent, as well as multi-categorical response models, where responses are not simply binary. To further name a few application examples in these contexts: by adapting the design to environmental stimuli, one could study the incentives at which individuals will cease to discount future environmental outcomes with a given probability (i.e., certainty). Translating the paradigm to different cognitive processes such as associative memory, one may aim at adjusting stimuli to homogenize associative memory traces or induce comparable learning speeds, which have been found to be highly heterogeneous (see e.g., Lonsdorf and Merz, 2017). Finally, in experiments of social interaction, one could even conceive of constructing artificial agents that follow individualized model-based behavioral suggestions which aim at inducing cooperative behavior in their interaction partner. This could for instance prove useful when training social skills or reducing negative biases in a therapeutic context.

## Conclusion

We propose a generic framework to manipulate and validate experimental conditions based on a specific class of interpretable behavioral latent variable models. These models may be leveraged to generate precise and falsifiable behavioral predictions which may be used to evoke graded and homogeneous choice probabilities. Statistical learning theory formally defines how to assess the degree of agreement between observations and predictions and thus how indicative observations are of the latent process, sometimes referred to as predictive validity (Yarkoni and Westfall, 2017). Assessing validity in terms of PEs in this context has a number of advantages. For one, since the PE may be used to uncover domains at which an instrument may fail to be valid, it may provide insights into how an instrument or model may be improved. Also, a low PE provides evidence for the latent process model itself, as experimental manipulations follow proposed hypotheses. As illustrated earlier, this paves the way to identify novel models, delineate differences to alternative models, or improve current models by model selection. Finally, improving validity in the above mentioned sense should help us homogenize behavior between participants, as a more valid experiment will generate more precise behavioral predictions by which participants may be grouped. The proposed approach can in principle be applied with little adaptation to other cognitive domains including learning and other types of decision making, as also outlined here.

## Data availability statement

The datasets presented in this study can be found in online repositories. The names of the repository/repositories and accession number(s) can be found below: https://github.com/GKoppe/data_code_repository_gradedDiscounting.

## Ethics statement

The studies involving human participants were reviewed and approved by Medical Faculty Mannheim, Heidelberg University (2019-633N). The participants provided their written informed consent to participate in this study.

## Author contributions

GK and PK conceptualized the study. GK, JT, MP, PK, and WS contributed to the design of the study. MP compiled the online experiment and collected the data. GK and JT performed the statistical analyses and wrote the manuscript. DD, GK, JT, MP, PK, and WS contributed to reading, revising, and approving the submitted manuscript. All authors contributed to the article and approved the submitted version.

## Footnotes

1
https://scipy.org/citing-scipy/

## References

[B1] Ahn W.-Y. Gu H. Shen Y. Haines N. Hahn H. A. Teater J. E. (2020). Rapid, precise, and reliable measurement of delay discounting using a Bayesian learning algorithm. *Sci. Rep.* 10:12091. 10.1038/s41598-020-68587-x 32694654 PMC7374100

[B2] Ainslie G. (1975). Specious reward: A behavioral theory of impulsiveness and impulse control. *Psychol. Bull.* 82 463–509. 10.1037/h0076860 1099599

[B3] Babor T. De La Fuente J. Saunders J. Grant M. (1992). *The alcohol use disorders identification test: Guidelines for use in primary health care.* Geneva: World Health Organization.

[B4] Bailey A. J. Romeu R. J. Finn P. R. (2021). The problems with delay discounting: A critical review of current practices and clinical applications. *Psychol. Med.* 51 1799–1806. 10.1017/S0033291721002282 34184631 PMC8381235

[B5] Batsikadze G. Diekmann N. Ernst T. M. Klein M. Maderwald S. Deuschl C. (2022). The cerebellum contributes to context-effects during fear extinction learning: A 7T fMRI study. *NeuroImage* 253:119080. 10.1016/j.neuroimage.2022.119080 35276369

[B6] Białaszek W. Marcowski P. Cox D. J. (2020). Comparison of multiplicative and additive hyperbolic and hyperboloid discounting models in delayed lotteries involving gains and losses. *PLoS One* 15:e0233337. 10.1371/journal.pone.0233337 32442186 PMC7244164

[B7] Bickel W. K. (2015). Discounting of delayed rewards as an Endophenotype. *Biol. Psychiatry* 77 846–847. 10.1016/j.biopsych.2015.03.003 25925716

[B8] Boslaugh S. (2012). *Statistics in a nutshell*, 2nd Edn. Sebastopol, CA: O’Reilly Media.

[B9] Cavagnaro D. R. Aranovich G. J. McClure S. M. Pitt M. A. Myung J. I. (2016). On the functional form of temporal discounting: An optimized adaptive test. *J. Risk Uncertain.* 52 233–254. 10.1007/s11166-016-9242-y 29332995 PMC5764197

[B10] Chakroborty P. Pinjari A. R. Meena J. Gandhi A. (2021). A psychophysical ordered response model of time perception and service quality: Application to level of service analysis at toll plazas. *Transp. Res. B Methodol.* 154 44–64. 10.1016/j.trb.2021.09.010

[B11] Cheng Y. S. Ko H. C. Sun C. K. Yeh P. Y. (2021). The relationship between delay discounting and Internet addiction: A systematic review and meta-analysis. *Addict. Behav.* 114:106751. 10.1016/j.addbeh.2020.106751 33310692

[B12] Collins A. G. Albrecht M. A. Waltz J. A. Gold J. M. Frank M. J. (2017). Interactions among working memory, reinforcement learning, and effort in value-based choice: A new paradigm and selective deficits in schizophrenia. *Biol. Psychiatry* 82 431–439. 10.1016/j.biopsych.2017.05.017 28651789 PMC5573149

[B13] Cox D. J. Dallery J. (2016). Effects of delay and probability combinations on discounting in humans. *Behav. Process.* 131 15–23. 10.1016/j.beproc.2016.08.002 27498073 PMC5441548

[B14] da Matta A. Gonçalves F. L. Bizarro L. (2012). Delay discounting: Concepts and measures. *Psychol. Neurosci.* 5 135–146. 10.3922/j.psns.2012.2.03

[B15] Dagher A. Owen A. M. Boecker H. Brooks D. J. (1999). Mapping the network for planning: A correlational PET activation study with the Tower of London task. *Brain* 122 1973–1987. 10.1093/brain/122.10.1973 10506098

[B16] Davison M. McCarthy D. (1988). *The matching law: A research review.* Hillsdale, NJ: Lawrence Erlbaum Associates, Inc.

[B17] Durstewitz D. Koppe G. Toutounji H. (2016). Computational models as statistical tools. *Curr. Opin. Behav. Sci.* 11 93–99. 10.1016/j.cobeha.2016.07.004

[B18] Ebert J. E. Prelec D. (2007). The fragility of time: Time-insensitivity and valuation of the near and far future. *Manage. Sci.* 53 1423–1438. 10.1287/mnsc.1060.0671 19642375

[B19] Efron B. (2021). Resampling plans and the estimation of prediction error (No. 4). *Stats* 4 1091–1115. 10.3390/stats4040063

[B20] Efron B. Hastie T. (2021). *Computer age statistical inference: Algorithms, evidence, and data science*, Vol. 6. Cambridge: Cambridge University Press. 10.1017/9781108914062

[B21] Estle S. J. Green L. Myerson J. Holt D. D. (2006). Differential effects of amount on temporal and probability discounting of gains and losses. *Mem. Cogn.* 34 914–928. 10.3758/BF03193437 17063921

[B22] Fechner G. T. (1858). Über ein wichtiges psychophysiches grundgesetz und dessen beziehung zur schäzung der sterngrössen. *Abk. K. Ges. Wissensch. Math. Phys. K1* 4, 455–532.

[B23] Frederick S. Loewenstein G. O’donoghue T. (2002). Time discounting and time preference: A critical review. *J. Econ. Lit.* 40 351–401. 10.1257/jel.40.2.351

[B24] Goodhew S. C. Edwards M. (2019). Translating experimental paradigms into individual-differences research: Contributions, challenges, and practical recommendations. *Conscious. Cogn.* 69 14–25. 10.1016/j.concog.2019.01.008 30685513

[B25] Green L. Fry A. F. Myerson J. (1994). Discounting of delayed rewards: A life-span comparison. *Psychol. Sci.* 5 33–36. 10.1111/j.1467-9280.1994.tb00610.x

[B26] Grinband J. Hirsch J. Ferrera V. P. (2006). A neural representation of categorization uncertainty in the human brain. *Neuron* 49 757–763. 10.1016/j.neuron.2006.01.032 16504950

[B27] Grosskopf C. M. Kroemer N. B. Pooseh S. Böhme F. Smolka M. N. (2021). Temporal discounting and smoking cessation: Choice consistency predicts nicotine abstinence in treatment-seeking smokers. *Psychopharmacology* 238 399–410. 10.1007/s00213-020-05688-5 33216166 PMC7826310

[B28] Hare T. A. Camerer C. F. Rangel A. (2009). Self-control in decision-making involves modulation of the vmPFC valuation system. *Science* 324 646–648. 10.1126/science.1168450 19407204

[B29] Hastie T. Tibshirani R. Friedman J. H. Friedman J. H. (2009). *The elements of statistical learning: Data mining, inference, and prediction*, Vol. 2. Berlin: Springer. 10.1007/978-0-387-84858-7

[B30] Hedge C. Powell G. Sumner P. (2018). The reliability paradox: Why robust cognitive tasks do not produce reliable individual differences. *Behav. Res. Methods* 50 1166–1186. 10.3758/s13428-017-0935-1 28726177 PMC5990556

[B31] Hurvich C. M. Tsai C.-L. (1989). Regression and time series model selection in small samples. *Biometrika* 76 297–307. 10.1093/biomet/76.2.297

[B32] Huys Q. J. Maia T. V. Frank M. J. (2016). Computational psychiatry as a bridge from neuroscience to clinical applications. *Nat. Neurosci.* 19 404–413. 26906507 10.1038/nn.4238 PMC5443409

[B33] Huys Q. J. Pizzagalli D. A. Bogdan R. Dayan P. (2013). Mapping anhedonia onto reinforcement learning: A behavioural meta-analysis. *Biol. Mood Anxiety Disord.* 3 1–16. 10.1186/2045-5380-3-12 23782813 PMC3701611

[B34] Insel T. Cuthbert B. Garvey M. Heinssen R. Pine D. S. Quinn K. (2010). Research domain criteria (RDoC): Toward a new classification framework for research on mental disorders. *Am. J. Psychiatry* 167 748–751. 10.1176/appi.ajp.2010.09091379 20595427

[B35] Jackson S. L. (2011). *Research methods and statistics: A critical thinking approach*, 4th Edn. Belmont, CA: Cengage Learning.

[B36] Kanai R. Rees G. (2011). The structural basis of inter-individual differences in human behaviour and cognition. *Nat. Rev. Neurosci.* 12 231–242. 10.1038/nrn3000 21407245

[B37] Keidel K. Rramani Q. Weber B. Murawski C. Ettinger U. (2021). Individual differences in intertemporal choice. *Front. Psychol.* 12:643670. 10.3389/fpsyg.2021.643670 33935897 PMC8085593

[B38] Knorr F. G. Neukam P. T. Fröhner J. H. Mohr H. Smolka M. N. Marxen M. (2020). A comparison of fMRI and behavioral models for predicting inter-temporal choices. *NeuroImage* 211:116634. 10.1016/j.neuroimage.2020.116634 32081783

[B39] Koffarnus M. N. Deshpande H. U. Lisinski J. M. Eklund A. Bickel W. K. LaConte S. M. (2017). An adaptive, individualized fMRI delay discounting procedure to increase flexibility and optimize scanner time. *NeuroImage* 161 56–66. 10.1016/j.neuroimage.2017.08.024 28803942 PMC5696088

[B40] Kohler R. J. Lichenstein S. D. Yip S. W. (2022). Hyperbolic discounting rates and risk for problematic alcohol use in youth enrolled in the Adolescent Brain and Cognitive Development study. *Addict. Biol.* 27:2. 10.1111/adb.13160 35229959 PMC9289942

[B41] Koppe G. Mallien A. S. Berger S. Bartsch D. Gass P. Vollmayr B. (2017). CACNA1C gene regulates behavioral strategies in operant rule learning. *PLoS Biol.* 15:e2000936. 10.1371/journal.pbio.2000936 28604818 PMC5467799

[B42] Koppe G. Meyer-Lindenberg A. Durstewitz D. (2021). Deep learning for small and big data in psychiatry. *Neuropsychopharmacology* 46 176–190. 10.1038/s41386-020-0767-z 32668442 PMC7689428

[B43] Kuha J. (2004). AIC and BIC: Comparisons of assumptions and performance. *Sociol. Methods Res.* 33 188–229. 10.1177/0049124103262065

[B44] Laibson D. (1997). Golden eggs and hyperbolic discounting. *Q. J. Econ.* 112 443–478. 10.1162/003355397555253

[B45] Leek M. R. (2001). Adaptive procedures in psychophysical research. *Percept. Psychophys.* 63 1279–1292. 10.3758/BF03194543 11800457

[B46] Levitt E. Sanchez-Roige S. Palmer A. A. MacKillop J. (2020). Steep discounting of future rewards as an impulsivity phenotype: A concise review. *Curr. Top. Behav. Neurosci.* 47 113–138. 10.1007/7854_2020_128 32236897

[B47] Loewenstein G. Prelec D. (1992). Anomalies in intertemporal choice: Evidence and an interpretation. *Q. J. Econ.* 107 573–597. 10.2307/2118482

[B48] Lonsdorf T. B. Merz C. J. (2017). More than just noise: Inter-individual differences in fear acquisition, extinction and return of fear in humans-Biological, experiential, temperamental factors, and methodological pitfalls. *Neurosci. Biobehav. Rev.* 80 703–728. 10.1016/j.neubiorev.2017.07.007 28764976

[B49] Mazur J. E. (1987). An adjusting procedure for studying delayed reinforcement. *Quant. Anal. Behav.* 5 55–73.

[B50] McKerchar T. L. Pickford S. Robertson S. E. (2013). Hyperboloid discounting of delayed outcomes: Magnitude effects and the gain-loss asymmetry. *Psychol. Rec.* 63 441–451. 10.11133/j.tpr.2013.63.3.003

[B51] Miedl S. F. (2012). Altered neural reward representations in pathological gamblers revealed by delay and probability discounting. *Arch. Gen. Psychiatry* 69 177–186. 10.1001/archgenpsychiatry.2011.1552 22310505

[B52] Monterosso J. R. Ainslie G. Xu J. Cordova X. Domier C. P. London E. D. (2007). Frontoparietal cortical activity of methamphetamine-dependent and comparison subjects performing a delay discounting task. *Hum. Brain Mapp.* 28 383–393. 10.1002/hbm.20281 16944492 PMC6871407

[B53] Mumford J. A. (2012). A power calculation guide for fMRI studies. *Soc. Cogn. Affect. Neurosci.* 7 738–742. 10.1093/scan/nss059 22641837 PMC3427872

[B54] O’Donoghue T. Rabin M. (2000). The economics of immediate gratification. *J. Behav. Decis. Mak.* 13 233–250. 10.1002/(SICI)1099-0771(200004/06)13:2<233::AID-BDM325>3.0.CO;2-U

[B55] Odum A. L. Baumann A. A. L. Rimington D. D. (2006). Discounting of delayed hypothetical money and food: Effects of amount. *Behav. Process.* 73 278–284. 10.1016/j.beproc.2006.06.008 16926071

[B56] Owen L. Browder J. Letham B. Stocek G. Tymms C. Shvartsman M. (2021). Adaptive nonparametric psychophysics. *arXiv* [Preprint]. Available online at: http://arxiv.org/abs/2104.09549 (accessed April 19, 2021).

[B57] Peters J. Miedl S. F. Büchel C. (2012). Formal comparison of dual-parameter temporal discounting models in controls and pathological gamblers. *PLoS One* 7:e47225. 10.1371/journal.pone.0047225 23226198 PMC3511467

[B58] Phelps E. Pollak R. (1968). On second-best national saving and game-equilibrium growth. *Rev. Econ. Stud.* 35 185–199. 10.2307/2296547 11010236

[B59] Pine A. Seymour B. Roiser J. P. Bossaerts P. Friston K. J. Curran H. V. (2009). Encoding of marginal utility across time in the human brain. *J. Neurosci.* 29 9575–9581. 10.1523/JNEUROSCI.1126-09.2009 19641120 PMC2816907

[B60] Pooseh S. Bernhardt N. Guevara A. Huys Q. J. M. Smolka M. N. (2018). Value-based decision-making battery: A Bayesian adaptive approach to assess impulsive and risky behavior. *Behav. Res. Methods* 50 236–249. 10.3758/s13428-017-0866-x 28289888

[B61] Prevost C. Pessiglione M. Metereau E. Clery-Melin M.-L. Dreher J.-C. (2010). Separate valuation subsystems for delay and effort decision costs. *J. Neurosci.* 30 14080–14090. 10.1523/JNEUROSCI.2752-10.2010 20962229 PMC6634773

[B62] Rabin M. O’Donoghue T. (1999). Doing it now or later. *American* 89 103–124. 10.1257/aer.89.1.103

[B63] Rachlin H. (2006). Notes on discounting. *J. Exp. Anal. Behav.* 85 425–435. 10.1901/jeab.2006.85-05 16776060 PMC1459845

[B64] Ratcliff R. (1978). A theory of memory retrieval. *Psychol. Rev.* 85 59–108. 10.1037/0033-295X.85.2.59

[B65] Ratcliff R. Rouder J. N. (1998). Modeling response times for two-choice decisions. *Psychol. Sci.* 9 347–356. 10.1111/1467-9280.00067

[B66] Rescorla R. A. Wagner A. R. (1972). “A theory of Pavlovian conditioning: Variations in the effectiveness of reinforcement and Nonreinforcement,” in *Classical conditioning II: Current research and theory*, eds Black A. H. Prokasy W. F. (New York, NY: Appleton- Century-Crofts), 64–99.

[B67] Ripke S. Hübner T. Mennigen E. Müller K. U. Li S.-C. Smolka M. N. (2014). Common neural correlates of intertemporal choices and intelligence in adolescents. *J. Cogn. Neurosci.* 27 387–399. 10.1162/jocn_a_00698 25208743

[B68] Ripke S. Hübner T. Mennigen E. Müller K. U. Rodehacke S. Schmidt D. (2012). Reward processing and intertemporal decision making in adults and adolescents: The role of impulsivity and decision consistency. *Brain Res.* 1478 36–47. 10.1016/j.brainres.2012.08.034 22940231

[B69] Rodzon K. Berry M. S. Odum A. L. (2011). Within-subject comparison of degree of delay discounting using titrating and fixed sequence procedures. *Behav. Process.* 86 164–167. 10.1016/j.beproc.2010.09.007 20932882 PMC3919556

[B70] Samuelson P. (1937). A note on measurement of utility. *Rev. Econ. Stud.* 4 155–161. 10.2307/2967612

[B71] Schmidt-Atzert L. Stefan K. Amelang M. (2021). “Grundlagen diagnostischer verfahren,” in *Psychologische diagnostik* (Berlin: Springer), 41–201.

[B72] Shamosh N. A. Gray J. R. (2008). Delay discounting and intelligence: A meta-analysis. *Intelligence* 36 289–305. 10.1016/j.intell.2007.09.004

[B73] Shen Y. Richards V. M. (2012). A maximum-likelihood procedure for estimating psychometric functions: Thresholds, slopes, and lapses of attention. *J. Acoust. Soc. Am.* 132 957–967. 10.1121/1.4733540 22894217 PMC3427362

[B74] Spinella M. (2007). Normative data and a short form of the Barratt Impulsiveness Scale. *Int. J. Neurosci.* 117 359–368. 17365120 10.1080/00207450600588881

[B75] Stevens S. (1957). On the psychophysical law. *Psychol. Rev.* 64 153–181. 10.1037/h0046162 13441853

[B76] Story G. W. Vlaev I. Seymour B. Darzi A. Dolan R. J. (2014). Does temporal discounting explain unhealthy behavior? A systematic review and reinforcement learning perspective. *Front. Behav. Neurosci.* 8:76. 10.3389/fnbeh.2014.00076 24659960 PMC3950931

[B77] Sutton R. S. Barto A. G. (2018). *Reinforcement learning: An introduction.* Cambridge, MA: MIT Press.

[B78] Taubert N. Christensen A. Endres D. Giese M. A. (2012). “Online simulation of emotional interactive behaviors with hierarchical Gaussian process dynamical models,” in *Proceedings of the 2012 ACM symposium on applied perception*, Los Angeles, CA, 25–32. 10.1145/2338676.2338682

[B79] Thomas M. L. Brown G. G. Patt V. M. Duffy J. R. (2021). Latent variable modeling and adaptive testing for experimental cognitive psychopathology research. *Educ. Psychol. Meas.* 81 155–181. 33456066 10.1177/0013164420919898 PMC7797961

[B80] Thome J. Pinger M. Halli P. Durstewitz D. Sommer W. H. Kirsch P. (2022). A model guided approach to evoke homogeneous behavior during temporal reward and loss discounting. *Front. Psychiatry* 13:846119. 10.3389/fpsyt.2022.846119 35800024 PMC9253427

[B81] van den Bos W. McClure S. M. (2013). Towards a general model of temporal discounting. *J. Exp. Anal. Behav.* 99 58–73. 10.1002/jeab.6 23344988 PMC8127626

[B82] Weber E. H. (1835). *De pulsu, resorptione, auditu et tactu annotationes anatomica et physiologica.* (Charleston, SC: Nabu Press), 152.

[B83] Wichmann F. A. Hill N. J. (2001). The psychometric function: I. Fitting, sampling, and goodness of fit. *Percept. Psychophys.* 63 1293–1313. 10.3758/BF03194544 11800458

[B84] Winer B. J. (1971). *Statistical principles in experimental design*, 2nd Edn. New York, NY: McGraw-Hill.

[B85] Wood G. Nuerk H.-C. Sturm D. Willmes K. (2008). Using parametric regressors to disentangle properties of multi-feature processes. *Behav. Brain Funct.* 4:38. 10.1186/1744-9081-4-38 18706088 PMC2535596

[B86] Yarkoni T. Westfall J. (2017). Choosing prediction over explanation in psychology: Lessons from machine learning. *Perspect. Psychol. Sci.* 12 1100–1122. 10.1177/1745691617693393 28841086 PMC6603289

