# Supplementary material for: Model-based experimental manipulation of probabilistic behavior in interpretable behavioral latent variable models
Source: Front Neurosci. 2023 Jan 9;16:1077735. doi: 10.3389/fnins.2022.1077735 (PMC9868576; doi:10.3389/fnins.2022.1077735)
Supplement: Supplementary file 1 [file Data_Sheet_1.pdf]

Supplementary Material

Methods

Table S1. Socio-demographic and subjective reports for the preliminary and the main experiment.

		Preliminary exp		Main exp	
		N=49		N=50	
age (mean/SD)		36.65	10.49	38.22	10.98
gender (N)	female	23		25	
	male	26		24	
	diverse	0		1	
education (N)	Primary	0		1	
	A-level	8		13	
	GCSE	6		6	
	Undergrad	26		20	
	Grad	9		8	
	PhD	0		2	
AUDIT-total (mean/SD)		3.98	3.27	5.16	5.45
BIS-total (mean/SD)		28.18	6.35	32.76	4.29
BIS-non-planning (mean/SD)		10.41	3.08	13.82	2.91
BIS-motor (mean/SD)		8.82	2.25	9.34	2.66
BIS-attentional (mean/SD)		8.96	2.47	9.6	1.92

Legend. AUDIT = Alcohol Use Disorder Identification Test, BIS = Barratt Impulsiveness Scale; exp = experiment; GCSE = general certificate of secondary education; Grad = graduate degree; Undergrad = undergraduate degree; SD = standard deviation

Table S2. Details on evaluated discounting models.

Name	Delayed value	Parameter constraints
hyperbolic model	$v_{del} = (\frac{1}{1 + \kappa \cdot D})r_{del}$	$\kappa \in [0, \infty)$
exponential model	$v_{del} = \kappa^D r_{del}$	$\kappa \in [0, 1]$
constant-sensitivity model	$v_{del} = exp(-(\kappa \cdot D)^{\delta})\, r_{del}$	$\kappa, \delta > 0$
modified hyperboloid model	$v_{del} = \frac{1}{(1 + \kappa \cdot D^s)}\, r_{del}$	$\kappa \in [0, \infty), s \in [0, 1]$
quasi-hyperbolic model	$v_{del} = \gamma\, \kappa^D r_{del}$	$\gamma, \kappa \in [0, 1]$
hyperboloid model	$v_{del} = \frac{1}{(1 + \kappa \cdot D)^s}\, r_{del},$	$\kappa \in [0, \infty), s \in [0, 1]$
double-exponential model	$v_{del} = (w\kappa_1{}^D + (1 - w)\kappa_2{}^D)r_{del}$	$w, \kappa_i \in [0, 1]$
Modified hyperboloid control model	same as modified hyperboloid model	$\beta = 1$

Results

Text S1. Effects of gender, age, and additional subjective measures.

Discounting behavior did not differ between gender (all p>0.162), or correlate with age (all p>0.332). The inferred model parameters s and κ did also not differ between gender (all p>0.322), or correlate with age (all p>0.122). We did observe higher choice parameters β in male as compared to female participants in run B (Z=2.91, p=0.004).

We further observed no correlations between any of the model parameters and subjectively reported alcohol use behavior or impulsivity (κ: all p_{corr}>0.44; s: all p_{corr}>0.44; β: all p_{corr}>0.055).

While the discounting parameter of run B (delay = 90) was positively associated with subjective impulsivity (BIS subscale: non-planning; r=.38, p_{corr}=0.036), we did not observe any further associations between subjective measurements and the discounting parameter in run A or in run B (all p>0.18).

Table S3. Socio-demographic information with respect to gender.

	**female**		**male**		**test-statistic**	
	N=25		N=24		Z	p
age (mean/SD)	37.92	10.98	38.83	11.31	0.28	0.779
**run A**						
% non-discounter	4		0			
imm choice freq (mean/SD)	53.39	11.77	55.97	20.31	0.24	0.810
explo-exploit par β (mean/SD)	1.59	1.87	1.31	3.35	0.95	0.342
discount par κ (mean/SD)	0.12	0.05	0.04	0.27	0.31	0.757
scaling par s (mean/SD)	0.71	0.28	0.67	0.32	0.19	0.845
discount factor (mean/SD)	0.67	0.20	0.73	0.29	0.05	0.960
**run B**						
% non-discounter	4		0			
imm choice freq (mean/SD)	45.06	15.80	38.27	18.87	1.40	0.162
explo-exploit par β (mean/SD)	0.59	27.17	13.85	0.89	2.91	0.004
discount par κ (mean/SD)	0.59	0.48	0.23	1.11	0.21	0.833
scaling par s (mean/SD)	0.44	0.36	0.55	0.41	0.99	0.322
discount factor (mean/SD)	0.64	0.24	0.63	0.30	0.33	0.741

Legend. discount = discounting; explo = exploration; exploit = exploitation; imm = immediate; freq = frequency; par = parameter; SD = standard deviation; % = percentage; please note, one individual has been excluded from the comparison as the individual did not identify as female or male

Text S2. Preliminary Experiment.

Rationale. The preliminary experiment was designed to test the hypothesis whether the proposed methodological framework can be used to resolve behavioral probabilities on a fine-grained, 9 level scale. In particular, we hypothesized that we can predict and induce discounting probabilities ranging from .1 to .9 in steps of .1 in a reward delay discounting task, using initial experimental settings as introduced earlier (Thome et al., 2022). The experiment is performed to provide a starting point for further improvements, and was then repeated and improved with the insights gained from the results reported here.

Sample. Fifty healthy participants completed the preliminary experiment (26 males, 24 females), with eligibility criteria as in the main experiment. One participant was excluded from further analyses due to not completing run B.

Experimental settings. In contrast to the experimental settings of the main experiment, the delayed rewards applied in the preliminary experiment were $r_{del} = \{5, 10, 20, 50\}$ £ (UK). Run A thus consisted of only 80 trials and run B of 180 trials. Everything else remained the same (cf. section 2.1).

Brief results report of preliminary experiment. ***Run A.*** In run A, we observed an average frequency of discounted choices of 55% (+/-20%) with only 2% of the sample showing less than 20% discounted choices. Supplementary Table S1 and S4 report sociodemographic information, subjective measurements, and gender effects. Test-retest reliability for the discount factors was high (evaluated for all delays: r>.7, p<.001), and moderate for β and κ (β: r=.42, p=.012; κ: r=.39, p=.037), but not the s parameter (r=.05, p=.76).

Table S4. Socio-demographic information of preliminary experiment with respect to gender.

	female		male		test-statistic	
	N=23		N=26		Z	*p*
age (mean/SD)	31.13	9.96	41.54	8.23	3.52	<0.001
**run A**						
% non-discounter	3.85		0			
imm choice freq (mean/SD)	50.71	16.37	58.04	24.04	0.92	0.356
explo-exploit par β (mean/SD)	10.74	25.16	9.84	28.35	0.81	0.417
discount par κ (mean/SD)	0.32	0.35	0.14	0.83	1.01	0.312
scaling par s (mean/SD)	0.75	0.29	0.72	0.36	0.97	0.331
discount factor (mean/SD)	0.61	0.29	0.59	0.34	0.47	0.638
**run B**						
% non-discounter	3.85		0			
imm choice freq (mean/SD)	38.15	24.98	44.65	20.32	0.70	0.483
explo-exploit par β (mean/SD)	5.97	3.71	2.31	20.77	0.47	0.638
discount par κ (mean/SD)	0.11	0.89	0.31	0.21	0.81	0.417
scaling par s (mean/SD)	0.71	0.37	0.62	0.36	1.39	0.164
discount factor (mean/SD)	0.67	0.29	0.66	0.33	0.61	0.541

Legend. discount = discounting; explo = exploration; exploit = exploitation; imm = immediate; freq = frequency; par = parameter; SD = standard deviation; % = percentage

Run B. While the GLM analysis confirmed a linear increase in observed discounting frequency with induced frequency (T(7)=13.81, *p*<.001), the slope deviated strongly from expectations (observed slope: .64 +/-.47, observed offset .1 +/-.31, predicted slope: .86, predicted offset .07, see Fig. S1A,B). We also observed a systematic increase in average PE in run B (see Fig. S1C).

On the single individual level, large PEs in run B were predicted by large (logarithmized) β parameters inferred on run A (r=.35, *p*=.018; Fig. S1D), as well as high log-likelihoods in run A (r=.89, *p*<.001). This is a sign of overfitting and would indicate that high β parameter estimates were in fact overestimated. This is supported by the observation that higher β parameters on run A deviated more strongly to those inferred on run B, that is, they were less reliably recovered (see Fig. S1E). We could not relate this bias to any particular behavioral pattern. Contrasting participants with low and high log-likelihoods in run A according to a median split revealed no differences in model parameters such as s and κ and discount factor (all *p*>0.242), or in direct behavioral measures such as immediate choice frequency and fraction of immediate to delayed responses (*p*>0.849). Also, none of these variables was predictive of PE in run B (all *p*>.13).

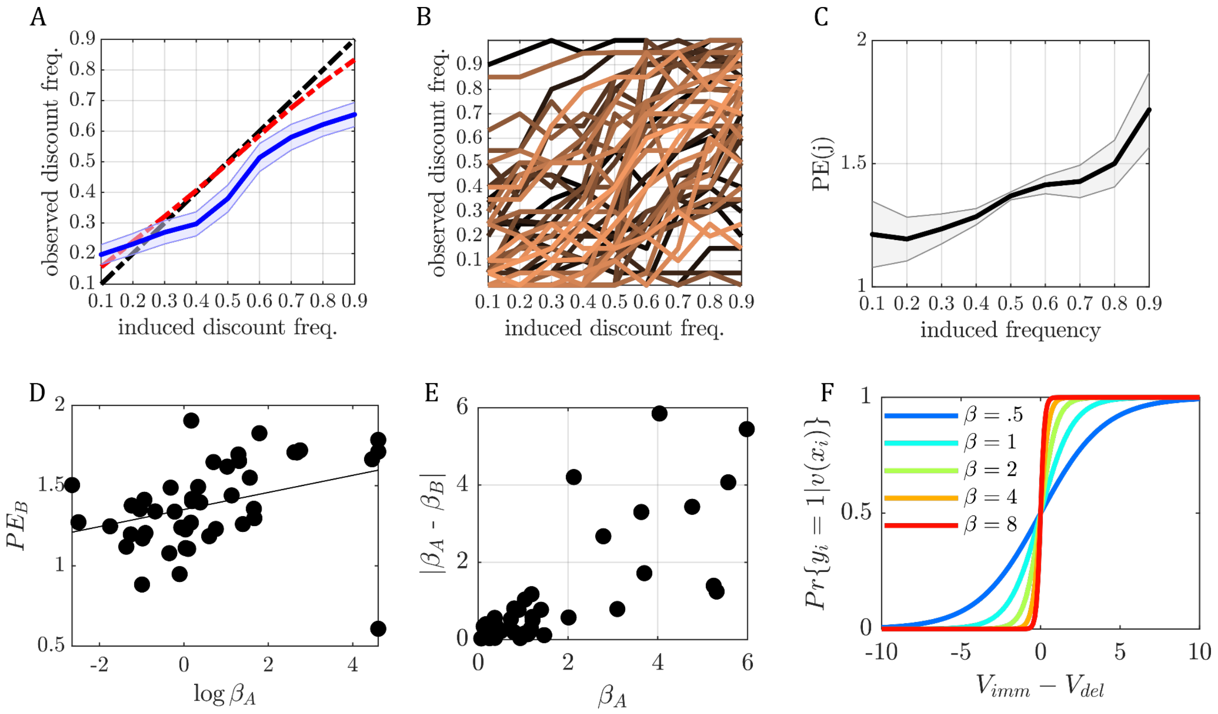

Fig. S1. Results of preliminary experiment. A. Relative frequency of discounted choices (y-axis) as a function of model induced frequencies (x-axis) averaged over all participants (mean and SEM are displayed). The black dashed line marks the identity, while the red dashed line shows the actual predicted frequencies according to the models. B. Single participant curves. C. Average prediction error (with SEM) as a function of experimental conditions. D. Correlation between inferred (logarithmized) β estimates of run A and PE assessed on run B. E. Inferred β estimates of run A (x-axis) plotted against the absolute difference between estimates of run A and B (y-axis). Larger β values indicate a higher deviation and thus a less reliable estimate. F. Effect of parameter β on transitioning between low and high immediate choice probabilities. Displayed are different hypothetical β values. For $\beta \geq 2$, transitions become very steep.

Comparison to main experiment. Comparing results to the main exp, the observed offset in the preliminary exp was higher and the slope lower (offset: T(97)=2.04, *p*=.044; slope: T(97)=-1.99, *p*=.049). Also, the average PE estimates obtained from run B were lower in the main exp compared to the preliminary exp (Z=3.49, *p*<.001).

Brief discussion of results and adaptations for main experiment. A large proportion of individuals (>60%) exhibited a continuous increase in discounting frequency coinciding with induced probabilities in line with model predictions. However, a fraction of individuals deviated from model predictions by exhibiting particularly large PEs in run B, low discounting slopes in run B, and high β estimates in run A. We found evidence that high β estimates may have been biased and overestimated.

Since the condition for run B trial generation (eqn. (3)) depends on β, a biased β will result in biased trials which will fail to induce the proposed discounting frequencies properly. Intuitively, β regulates the sensitivity by which an individual distinguishes small differences in value (cf. eqn. (2); see Fig. S1F). In the trial-generating condition, as $\beta \rightarrow \infty$, the right side of the equation approaches 0 (i.e., $\frac{log(\frac{p_{imm}}{1-p_{imm}})}{\beta} \rightarrow 0$), such that generated trials will move closer to the indifference point (where $v_{imm} = v_{del}$; see also Fig. S1F). More sensitive individuals will therefore require smaller differences to discriminate between immediate and delayed rewards. However, if β is overestimated, the choice between these options will simply become more difficult (Fig. S1F). Biased β estimates therefore account for the high PEs observed in a fraction of the individuals.

As behavior becomes more conform with the model (and β increases), it becomes increasingly difficult to infer β. For high β values the probability function converges towards a delta function, and consistent choices with probability close to 1 have little impact on the log-likelihood. In other words, the log-likelihood function does not discriminate well between β values for model consistent behavior. In short time series, it is more likely to obtain model consistent behavior by chance, resulting in overestimation of this parameter.

For the main experiment, we therefore decided on improving the β estimation by 1) increasing the number of trials in run A from 80 to 100 (by adding an additional delayed outcome of 100) and 2) constraining β to lie within .001 and 2. We reasoned that increasing the number of trials should improve the discrimination of parameter estimates (Winer et al., 1971). Also, at $\beta \geq 2$, we started to observe low test-retest reliability (Fig. S1E), and higher values result in similarly steep slopes (Fig. S1F).

Text S3. References

Winer, B. J., Brown, D. R., & Michels. (1971). *Statistical Principles in Experimental Design* (2nd ed.). McGraw-Hill Publishing Co.
